# Effects of home visits on quality of life among older adults: a systematic review protocol

**DOI:** 10.1186/s13643-021-01862-8

**Published:** 2021-12-06

**Authors:** Yea Lu Tay, Nurul Salwana Abu Bakar, Ruzimah Tumiran, Noor Hasidah Ab Rahman, Noor Areefa Ameera Mohd Ma’amor, Weng Keong Yau, Zalilah Abdullah

**Affiliations:** 1 Institute for Health Systems Research, National Institutes of Health, Ministry of Health Malaysia, 40170 Shah Alam, Selangor Malaysia; 2 Institute of Biological Sciences, Faculty of Science, Universiti Malaya, 50603 Kuala Lumpur, Malaysia; 3 General Medical Department, Hospital Kuala Lumpur, Ministry of Health Malaysia, Jalan Pahang, 50586 Kuala Lumpur, Malaysia

**Keywords:** Systematic review, Meta-analysis, House calls, Home visits, Aged

## Abstract

**Background:**

Home visiting services for older adults have been offered for decades to maintain and promote health and independent functioning, thus enhancing quality of life. Previous systematic reviews have provided a mixed picture of the benefits of home visiting programmes in older adults, primarily because of heterogeneity in study designs, targeted populations, and intervention strategies. These reviews may also become out of date; thus, an updated synthesis of relevant studies is warranted. Our objective is to perform a systematic review of recently published primary studies on the effectiveness of multi-professional home visits on quality of life among older adults.

**Methods:**

We will perform a comprehensive search for studies investigating the effect of a multi-professional home visit approach on quality of life among older adults. We will conduct the literature search in selected electronic databases and relevant research websites from January 2010 onwards. We will include randomised controlled trials (RCTs), cluster randomised controlled trials (cluster RCTs), and observational studies that enrolled older adults without dementia over 60 years old, along with studies involving multi-professional preventive–promotive home visit approaches not related to recent hospital discharge. We will report our planned review following the Preferred Reporting Items for Systematic Reviews and Meta-Analyses (PRISMA) guidelines. We will retrieve and record relevant data in a standardised data extraction form and evaluate the quality of the included articles using the Cochrane risk of bias tool and the quality assessment tool for studies with diverse designs (QATSDD). Where appropriate, outcomes will be pooled for meta-analysis using a random-effects model. The main outcomes include quality of life, incidence of falls, depression, dementia, and emergency department admissions.

**Discussion:**

This review may provide evidence for the effectiveness of home visits in improving older adults’ quality of life. It will potentially benefit health care professionals, policymakers, and researchers by facilitating the design and delivery of interventions related to older generations and improve service delivery in future.

**Systematic review registration:**

PROSPERO CRD42021234531.

**Supplementary Information:**

The online version contains supplementary material available at 10.1186/s13643-021-01862-8.

## Background

Population ageing is a global phenomenon. Most individuals expect to live into their sixties and beyond. The world population of adults aged 60 years and over is expected to nearly double from 12 to 22% between 2015 and 2050 [1]. In Malaysia, 20% of the total population will be over 65 years old by 2030 [2]. A recent study indicated that the life expectancy of Malaysian older adults aged 65 years was 79.8 and 82.1 years for males and females, respectively [3]. Despite the national census statistics defining older adults as those over the age of 65, Malaysia adopted the United Nations’ definition, classifying older adults as those aged 60 years and above for policy development regarding the older adult population [4]. The older adult population has a multitude of health needs and challenges, along with a deteriorating quality of life (QoL) [5].

According to the World Health Organization (WHO), QoL refers to “an individual’s perception of life in the context of the culture and value system in which he or she lives and in relation to his or her goals, expectations, standards, and concerns” [6]. QoL linked to health concepts is defined as the value assigned to the duration of life, modulated by limitations, functional status, perceptions, and social opportunities, which are influenced by diseases, injuries, treatments, and health policies [7]. QoL is increasingly recognised as a focus for healthcare service delivery in the older adult population. It allows the healthcare providers and policymakers to measure the efficacy of health interventions and evaluate multi-sectoral public policies, which include health, social, community, and policy actions [8].

Numerous healthcare interventions have been designed and implemented with the goal of maintaining or improving QoL among older adults, and most studies indicate the importance of active ageing. These studies have demonstrated that QoL among older adults can be enhanced through low-cost interventions, such as physical exercise [9–11]. Besides, older adults utilising the home visiting services were shown to have a better QoL outcome [12, 13].

Home visits are defined as visits to an individual’s home by professionals, which may include nurses, social workers, physicians, physiotherapists, occupational therapists, pharmacists and other specialists [14]. There are five types of home visiting services: palliative, rehabilitative, long-term maintenance, therapeutic, and preventive–promotive home visits [15]. Preventive–promotive home visiting services have been offered for decades with the goal of maintaining and promoting the health and independent functioning of older adults. In addition, these services aim to reduce admission to hospitals or nursing homes and the associated economic burden [16, 17].

Home visits allow health professionals to evaluate possible problems in the living environment of homebound older adults, assess their physical and mental health status, provide older adults with professional support, and refer them to specialist care if needed [17]. By reducing the risk of functional deterioration, these strategies are primarily structured to enhance the health-related QoL (HRQoL) of older adults, increase the possibility of continued independent living, and delay mortality [18].

Home visits have been shown to positively affect patient care and provider attitudes as well as increased satisfaction among homebound older adults and providers [19]. A previous study demonstrated that preventive home visits may have positive effects on QoL of older adults [20]. However, the variability in the study designs, participants, and outcome measures has made comparisons difficult. Liimata et al. (2019) conducted a randomised controlled trial (RCT) measuring the effects of preventative multidisciplinary home visits on HRQoL of older adults living independently. The team, which consisted of a nurse, a physiotherapist, and a social worker, observed a significantly slower decline of HRQoL in the intervention group, but this effect diminished after the visits ended [20]. In a separate publication from the same study, preventive home visits resulted in an improved HRQoL without incurring additional healthcare costs [21]. An effective prevention method aids in supporting quality of life among older adults. In a review on preventive home visits for older adults, Mayo-Wilson et al. (2014) analysed 64 RCTs involving older adults without dementia from database inception until December 2012. The study yielded high-quality evidence for decreasing falls but low-quality evidence for quality of life [22]. Thus, although an RCT demonstrated promising results on home visits, a review of multiple RCTs failed to observe significant results. In addition, although multi-professional preventive home visit approaches with thorough evaluation and collaboration among healthcare professionals may be more beneficial than home visits by a single professional, few studies have focused on this multi-professional preventive home visit approach [20, 23, 24].

Multi-professional preventive home visit interventions involve coordination between several health care professionals towards shared goals. Effective communication among the team members is crucial when the members work within the boundaries of their expertise and subsequently discuss progress in group sessions [25]. Previous systematic reviews have provided a mixed picture of the benefits of multi-professional home visiting services for older adults. Stuck et al. [26] and Touringy et al. [14] suggested that the multi-professional approach with follow-up visits was effective in identifying the needs of the older adult population. However, Mayo-Wilson et al. [22] demonstrated the challenges of concluding that preventive home visits result in reliable benefits, primarily due to variability in the study designs, participants, and intervention strategies of the preventive home visits approach.

### Rationale

In Malaysia, home visiting services for the older adult population are delivered by a multidisciplinary team and are primarily provided by the Ministry of Health [27]. The home visiting services offered in Malaysia include home-based treatment, pharmacy counselling, rehabilitation, and palliative services, which aim to ensure continuity of care at home, reduce hospital readmission, and improve QoL [28, 29]. According to the National Health and Morbidity Survey (NHMS) 2018, a national community survey for elderly health in Malaysia, 28.6% of older adults perceived themselves as having poor QoL, 14.1% reported having at least one fall in the 12 months prior to the survey, 8.5% were diagnosed with dementia, and 11.2% were at risk of experiencing depressive symptoms [30]. Poor QoL in Malaysian older adults was found to be associated with lower education, depression, food insecurity, reduced functional status, and a lack of social support [31]. Hence, we seek to examine preventive–promotive strategies that specifically prevent or reduce the risk of developing dementia, depression, and falls, with the ultimate aim of improving QoL among the older adult population.

To our knowledge, the most recent systematic review of primary studies examining the multi-professional preventive home visit approach for older adults included studies conducted up to December 2012 [22]. Because the older adult population is rapidly growing, the number of studies describing the home visit intervention is increasing, and the methodological and reporting quality of these studies is improving. Hence, a comprehensive systematic review which includes recent studies is needed to provide new evidence on the effectiveness of multi-professional preventive–promotive home visits in improving QoL among older adults. This review may serve as a guideline for the healthcare professionals, policymakers, researchers, and institutions in designing and delivering interventions for older adults in future. Aligning health systems with the needs of the older adult population may help to promote healthy ageing in Malaysia in the long term.

### Objective

This study aims to systematically assess the effect of a multi-professional home visit approach on QoL among older adults.

## Methods

The present protocol has been registered within the PROSPERO database (registration number CRD42021234531) and is being reported in accordance with the reporting guidance provided in the Preferred Reporting Items for Systematic Reviews and Meta-Analyses Protocols (PRISMA-P) statement [32, 33] (see checklist in Additional file 1).

### Eligibility criteria

#### Types of studies

Randomised controlled trials (RCTs), cluster randomised controlled trials (cluster RCTs), and observational studies (such as cohort, case-control, and cross-sectional studies) will be included. Quasi-randomised controlled trials (quasi-RCTs), which are often associated with a high risk of bias, and cross-over studies will be excluded. Case reports, guidelines, protocols, and short communication will also be excluded.

#### Population

We will only include studies examining the older adults without dementia aged 60 years and above who reside in their own homes and receive treatment at primary care outpatient departments. We will exclude studies that involve older adults living in retirement homes or nursing homes.

#### Types of interventions

We will include studies that aim specifically to assess the following interventions:

1. Home visits which aim to prevent or reduce risks related to ageing
2. Home visits which utilise at least two of the following multidimensional approaches: medical, functional, psychosocial, and environmental evaluation of problems and resources, resulting in specific recommendations for solving observed problems and preventing new ones.

#### Types of outcome measures

##### Primary outcomes

We will measure QoL using validated scales such as the WHO QoL Questionnaires, WHOQoL-BREF [34] and WHOQoL-OLD [35], the 19-item Control, Autonomy, Self-Realisation and Pleasure (CASP-19) questionnaire [36], the Older People’s Quality of Life (OPQoL) questionnaire [37], and the 36-item Short Form Health Survey (SF-36) [38, 39].

##### Secondary outcomes

We will also analyse the effects of home visit interventions on the incidence of falls, depression, dementia, and emergency department admissions.

### Exclusion criteria

We will exclude studies that involve follow-up visits for recent hospital discharge and studies targeting people with one specific illness.

### Information sources

A comprehensive systematic electronic search will be conducted using these databases: PubMed, Ovid MEDLINE (R), the Cochrane Library, EMBASE, the Cochrane Central Register of Controlled Trials (CENTRAL), the Cumulative Index to Nursing and Allied Health Literature (CINAHL), Web of Science, ClinicalTrials.gov, the metaRegister of Controlled Trials, the Turning Research into Practice (TRIP) database, Open Grey, High Wire, the National Institute for Health and Care Excellence (NICE), and the National Institutes of Health (NIH). The search will be limited to English language articles published from January 2010 onwards.

In addition, cross-referencing will be performed, whereby the reference lists of articles will be scanned for relevant studies. We will hand-search Malaysian quality initiative or health systems project reports in the libraries of the Institute for Medical Research (IMR), Institute for Health Management (IHM), Institute for Health System Research (IHSR), Institute for Public Health (IPH), and Ministry of Health, Malaysia.

### Search strategy

The search strategy will be based on the key components of the research question: population, interventions, and outcomes. It will include a mix of medical subject headings (MeSH) terms and free-text terms in the title and abstract search fields of the databases. The keywords will be related to the participants (e.g., aged, senior, older, elder, and geriatric), home care (e.g., house calls, home visits, and home care), and the outcomes (e.g., quality of life and accidental falls). Examples of the search strategy are presented in Additional file 2.

### Selection of studies

Two review authors will examine the titles and abstracts independently and will exclude all irrelevant studies. Two review authors will independently retrieve and screen the full text of potentially relevant articles and identify those that meet the eligibility criteria. These steps will be recorded in an Excel table along with the reasons for study exclusion. To avoid duplication, data will be identified from the main source. Any disagreements that arise will be resolved through discussions with a third author. A PRISMA flow chart showing details of studies included and excluded at each stage of the study selection process will be provided [33].

### Data extraction

Two reviewers will independently retrieve and record data in a data extraction form. Any disagreements will be resolved through discussion with the third reviewer. The data extraction form will include the following variables:

- General information: title, first author, publication year, and country
- Methods: study design, study duration, sample size, and mean age of the sample
- Types of intervention: visitors’ professional group, number of visits, length of visits
- Outcome measures:
  - ○ Primary outcome: QoL (characteristics of the scales used to measure QoL)
  - ○ Secondary outcomes: incidence of falls, depression, dementia, and emergency department admissions

### Quality assessment

Two reviewers will evaluate the possible risk of bias for each study independently. Any disagreements will be discussed with the third reviewer. We will evaluate the RCT and cluster RCT articles for the methodological quality using the Cochrane risk of bias tool (RoB 2.0) [40]. We will categorise the risk of bias as low, high, or unclear in each of the following domains: allocation concealment, random sequence generation, blinding of outcome assessment, selective outcome reporting, incomplete outcome data, and other sources of bias.

The quality assessment tool for studies with diverse designs (QATSDD) [41, 42] will be utilised to assess mixed-method studies. There are 14 QATSDD evaluative indicators for quantitative studies. Each indicator will be measured on a 4-point Likert scale as follows: 0 (not at all), 1 (very slightly), 2 (moderate), and 3 (complete). The maximum score of this tool is 42. The quality of a study is rated as ‘high’ if the score is over 75%, ‘good’ if it is between 50 and 75%, ‘moderate’ if it is between 25 and 50%, and ‘poor’ if it is below 25%.

### Data synthesis and analysis

If the studies are sufficiently homogenous in terms of population, interventions, and outcomes, the results will be pooled, and a meta-analysis using a random-effects model will be conducted. Where possible, dichotomous data will be presented as relative risks (RRs) with 95% confidence intervals (CIs). Continuous data will be expressed as mean differences (MDs) or standardised mean differences (SMDs) (when the outcome is measured using several scales or instruments) with 95% CIs [43]. If the study characteristics are substantially different, the results will be analysed in the following subgroups, if data are available:

- Participant’s age: 60–79, ≥80
- Visitors’ professional group

We will interpret the heterogeneity and variability of the included studies in relation to population, interventions, outcomes, and methods. When meta-analysis is attempted, heterogeneity will be evaluated by forest plots to assess whether the CIs overlap. In addition, heterogeneity among the included studies will be measured using the chi-square (*χ*^2^) test and *I*^2^ statistic. A small *p* value (*p* < 0.1) for the *χ*^2^ test and an *I*^2^ of 50% or higher indicate moderate to substantial heterogeneity [44].

If meta-analysis is not possible, a narrative will be developed to summarise differences. We will present the data in a summary table outlining the content of the included primary studies (the number of participants, study population, description of interventions), as well as the results, conclusions, and quality ranking of studies.

### Meta-bias(es)

We will assess publication bias using the Tandem method. If possible, the potential for reporting bias will be further explored using a funnel plot. A linear regression test will be performed to examine the degree of publication bias. Publication bias is significant if the p-value is less than 0.1.

### Confidence in cumulative evidence

The quality of the evidence synthesised in this review will be assessed using the Grading of Recommendations Assessment, Development and Evaluation (GRADE) methodology [45]. This methodology involves the evaluation of the evidence quality for each outcome across the domains of risk of bias, consistency, directness of evidence, precision of effect estimates, and publication bias, resulting in the following grades for each outcome: high, moderate, low, or very low [17, 46].

## Discussion

This review may serve as evidence to support effective interdisciplinary home visits that can improve health-related QoL among older adults. This will potentially benefit policymakers and healthcare managers in planning for an efficient resource utilisation and evidence-based policy designs catered to older adults’ health. Healthcare professionals and implementers will be able to deliver health programmes and interventions suited to the needs of the older adult population. Researchers and other institutions will gain knowledge of multiple health interventions. In addition, recognising international practices will provide information to policymakers regarding strategies to improve quality of care in future.

This review has potential limitations. Our search strategy may miss sources of information available in languages other than the English language. In addition, we anticipate that the review will face challenges due to the heterogeneous nature of the study design, particularly in interventions and outcomes measures, which may limit the interpretability and comparability of results.

### Protocol amendments

Any amendments to this protocol in the carrying out of this systematic review will be documented and reported in both the PROSPERO register and any subsequent publications.

### Dissemination plans

The findings of this systematic review will be disseminated through publication in peer-reviewed journals and via relevant conferences. In addition, the results will also be shared with potential stakeholders, such as the Ministry of Women, Family and Community Development and the Family Health Development Division under the Ministry of Health Malaysia.

## Supplementary Information

**Additional file 1:.** PRISMA 2020 Checklist

**Additional file 2:.** Search strategy

## Data Availability

Not applicable.

## Abbreviations

CI: Confidence interval
Cluster RCTs: Cluster randomised controlled trials
HRQoL: Health-related quality of life
MeSH: Medical subject headings
PRISMA: Preferred Reporting Items for Systematic Reviews and Meta-Analyses
QATSDD: Quality assessment tool for studies with diverse design
QoL: Quality of life
Quasi-RCTs: Quasi-randomised controlled trials
RCTs: Randomised controlled trials
WHO: World Health Organization
