# Supplementary material for: Effects of home visits on quality of life among older adults: a systematic review protocol
Source: Syst Rev. 2021 Dec 6;10:307. doi: 10.1186/s13643-021-01862-8 (PMC8650292; doi:10.1186/s13643-021-01862-8)
Supplement: Supplementary file 2 — Additional file 2:. Search strategy [file 13643_2021_1862_MOESM2_ESM.docx]

**ADDITIONAL FILE 2: SEARCH STRATEGY**

Table 1: PubMed search strategy

| **Search ID** | **SEARCH** |
| --- | --- |
| #1 | Geriatric assessment[MeSH Terms] OR geriatric*[Title/Abstract] |
| #2 | Aged[MeSH Terms] OR aged[Title/Abstract] OR elder*[Title/Abstract] OR olde*[Title/Abstract] OR senior*[Title/Abstract] OR aged 60+ years[Title/Abstract] |
| #3 | #1 OR #2 |
| #4 | House calls[MeSH Terms] OR home care services[MeSH Terms] OR home care agencies[MeSH Terms] OR home health aides[MeSH Terms] |
| #5 | Community health nursing[MeSH Terms] OR home health nursing[MeSH Terms] OR home care*[Title/Abstract] OR homecare[Title/Abstract] OR home support*[Title/Abstract] OR domiciliary care*[Title/Abstract] OR domiciliary visit*[Title/Abstract] OR domiciliary support*[Title/Abstract] OR domiciliary service*[Title/Abstract] OR "home health care"[Title/Abstract] OR home health agenc*[Title/Abstract] OR health visitor*[Title/Abstract] OR house call*[Title/Abstract] OR visiting nurse*[Title/Abstract] OR nurse visit*[Title/Abstract] OR "medical home"[Title/Abstract] OR home nurs*[Title/Abstract] OR "home based care"[Title/Abstract] OR mobile health unit*[Title/Abstract] OR "home rehabilitation"[Title/Abstract] OR "hospital at home"[Title/Abstract] |
| #6 | "Home based"[Title/Abstract] OR “in-home”[Title/Abstract] OR home visit*[Title/Abstract] |
| #7 | Rehab*[Title/Abstract] OR therap*[Title/Abstract] OR treatment[Title/Abstract] OR testing[Title/Abstract] OR health care provider*[Title/Abstract] OR health personnel[MeSH Terms] OR “health care personnel”[Title/Abstract] OR health professional*[Title/Abstract] OR health care professional*[Title/Abstract] OR healthcare professional*[Title/Abstract] OR physician*[Title/Abstract] OR psycholog*[Title/Abstract] OR psychiatr*[Title/Abstract] OR social work*[Title/Abstract] OR pharmac*[Title/Abstract] OR nutritionist*[Title/Abstract] OR speech-language pathologist*[Title/Abstract] OR patient care team[MeSH Terms] OR case management[MeSH Terms] OR patient care team*[Title/Abstract] OR case manage*[Title/Abstract] OR liaison nurse*[Title/Abstract] OR collaborative practice*[Title/Abstract] OR “collaborative care”[Title/Abstract] OR “nurse-led follow-up”[Title/Abstract] OR “interdisciplinary care”[Title/Abstract] OR interdisciplinary team*[Title/Abstract] OR interdisciplinary care team*[Title/Abstract] OR “multidimensional care”[Title/Abstract] OR multidimensional team*[Title/Abstract] OR multidimensional care team*[Title/Abstract] OR multi dimensional care[Title/Abstract] OR multi dimensional team*[Title/Abstract] OR multi dimensional care team*[Title/Abstract] OR “team care”[Title/Abstract] OR “multi agency working”[Title/Abstract] OR “inter agency working”[Title/Abstract] OR “multi professional working”[Title/Abstract] OR “interprofessional working”[Title/Abstract] OR interprofessional team*[Title/Abstract] OR inter professional team*[Title/Abstract] OR multidisciplinary team* [Title/Abstract] OR multidisciplinary care team*[Title/Abstract] OR interdisciplinary treatment approach*[Title/Abstract] OR "integrated care"[Title/Abstract] |
| #8 | #6 AND #7 |
| #9 | #4 OR #5 OR #8 |
| #10 | Quality of life[MeSH Terms] OR quality of life[Title/Abstract] OR QoL[Title/Abstract] |
| #11 | Accidental falls[MeSH Terms] OR fall*[Title/Abstract] |
| #12 | Depression[MeSH Terms] OR depress*[Title/Abstract] |
| #13 | Dementia[MeSH Terms] OR dementia*[Title/Abstract] |
| #14 | Emergency service, hospital[MeSH Terms] |
| #15 | #10 OR #11 OR #12 OR #13 OR #14 |
| #16 | Randomized controlled trials as topic[MeSH Terms] OR randomi* controlled trial*[Title/Abstract] OR RCT[Title/Abstract] OR RCTs[Title/Abstract] OR random allocation[MeSH Terms] OR controlled clinical trials as topic[MeSH Terms] OR clinical trials as topic[MeSH Terms] OR cohort studies[MeSH Terms] OR case-control studies[MeSH Terms]OR cross-sectional studies[MeSH Terms] |
| #17 | Systematic review[Publication Type] OR systematic review[Title] OR meta-analysis[Publication Type] OR meta analysis[Title] OR review[Publication Type] OR review[Title] OR letter[Publication Type] OR case reports[Publication Type] OR guideline[Publication Type] |
| #18 | #16 NOT #17 |
| #19 | #3 AND #9 AND #15 AND #18 |
| #20 | (("2010/01/01"[Date - Publication] : "2020/07/31"[Date - Publication])) AND (English[Language]) |
| #21 | #19 AND #20 |

#

# Table 2: CINAHL Complete search strategy

| **Search ID** | **Query** |
| --- | --- |
| S1 | (MH "Geriatric assessment+") OR TI geriatric* OR AB geriatric* |
| S2 | (MH "Aged+") OR TI (aged OR elder* OR old* OR senior* OR aged 60+ years) OR AB (aged OR elder* OR old* OR senior* OR aged 60+ years) |
| S3 | S1 OR S2 |
| S4 | (MH "Home visits") OR (MH "home health care+") OR (MH "home health agencies") OR (MH "home care equipment and supplies") OR (MH "home health aides") |
| S5 | (MH "Community health nursing+") OR (TI (Home care* OR homecare OR home support* OR domiciliary care* OR domiciliary visit* OR domiciliary support* OR domiciliary service* OR "home health care" OR home health agenc* OR health visitor* OR house call* OR visiting nurse* OR nurse visit* OR "medical home" OR home nurs* OR "home based care" OR mobile health unit* OR "home rehabilitation" OR "hospital at home") OR AB (Home care* OR homecare OR home support* OR domiciliary care* OR domiciliary visit* OR domiciliary support* OR domiciliary service* OR "home health care" OR home health agenc* OR health visitor* OR house call* OR visiting nurse* OR nurse visit* OR "medical home" OR home nurs* OR "home based care" OR mobile health unit* OR "home rehabilitation" OR "hospital at home")) |
| S6 | TI ("home based" OR “in-home” OR home visit*) OR AB ("home based" OR “in-home” OR home visit*) |
| S7 | ( (MH "Health personnel+") OR (MH "multidisciplinary care team+") OR (MH "case management") ) OR TI ( rehab* OR therap* OR treatment OR health care provider* OR “health care personnel” OR health professional* OR health care professional* OR healthcare professional* OR physician* OR psycholog* OR psychiatr* OR pharmac* OR nutritionist* OR speech-language pathologist* OR patient care team* OR case manage* OR liaison nurse* OR collaborative practice* OR “collaborative care” OR “nurse-led follow-up” OR “interdisciplinary care” OR interdisciplinary team* OR interdisciplinary care team* OR “multidimensional care” OR multidimensional team* OR multidimensional care team* OR multi dimensional care OR multi dimensional team* OR multi dimensional care team* OR “team care” OR “multi agency working” OR “inter agency working” OR “multi professional working” OR “interprofessional working” OR interprofessional team* OR inter professional team* OR multidisciplinary team* OR multidisciplinary care team* OR interdisciplinary treatment approach* OR "integrated care" ) OR AB ( rehab* OR therap* OR treatment OR health care provider* OR “health care personnel” OR health professional* OR health care professional* OR healthcare professional* OR physician* OR psycholog* OR psychiatr* OR pharmac* OR nutritionist* OR speech-language pathologist* OR patient care team* OR case manage* OR liaison nurse* OR collaborative practice* OR “collaborative care” OR “nurse-led follow-up” OR “interdisciplinary care” OR interdisciplinary team* OR interdisciplinary care team* OR “multidimensional care” OR multidimensional team* OR multidimensional care team* OR multi dimensional care OR multi dimensional team* OR multi dimensional care team* OR “team care” OR “multi agency working” OR “inter agency working” OR “multi professional working” OR “interprofessional working” OR interprofessional team* OR inter professional team* OR multidisciplinary team* OR multidisciplinary care team* OR interdisciplinary treatment approach* OR "integrated care") |
| S8 | S6 AND S7 |
| S9 | S4 OR S5 OR S8 |
| S10 | (MH "Quality of life+") OR TI (Quality of life OR QoL ) OR AB (Quality of life OR QoL ) |
| S11 | (MH "Accidental falls") OR TI fall* OR AB fall* |
| S12 | (MH "Depression+") OR TI depress* OR AB depress* |
| S13 | (MH "Dementia+") OR TI dementia* OR AB dementia* |
| S14 | (MH "Emergency service") |
| S15 | S10 OR S11 OR S12 OR S13 OR S14 |
| S16 | ( (MH "Randomized controlled trials+") OR (MH "random assignment") OR (MH "clinical trials+") OR (MH "prospective studies+") OR (MH "case control studies+") OR (MH "cross sectional studies") ) OR TI (randomi* controlled trial* OR RCT OR RCTs ) OR AB (randomi* controlled trial* OR RCT OR RCTs) |
| S17 | PT (Systematic review OR meta-analysis OR review OR letter OR case reports OR guideline) OR TI (systematic review OR meta-analysis OR review) |
| S18 | S16 NOT S17 |
| S19 | S3 AND S9 AND S15 AND S18 |

Table 3: Ovid MEDLINE (R) search strategy

| **Search ID** | **Query** |
| --- | --- |
| 1 | Geriatric assessment/ or geriatric*.tw. |
| 2 | exp Aged/ or (aged or elder* or older* or senior* or aged 60+ years).tw. |
| 3 | 1 or 2 |
| 4 | House calls/ or exp home care services/ or home care agencies/ |
| 5 | exp Community health nursing/ or home health nursing/ or (home care* or homecare or home support* or domiciliary care* or domiciliary visit* or domiciliary support* or domiciliary service* or home health care or home health agenc* or health visitor* or house call* or visiting nurse* or nurse visit* or "medical home" or home nurs* or home based care or mobile health unit* or home rehabilitation or hospital at home).tw. |
| 6 | (Home based or in-home or home visit*).tw. |
| 7 | exp Health personnel/ or exp patient care team/ or case management/ or (rehab* or therap* or treatment or health care provider* or health care personnel or health professional* or health care professional* or healthcare professional* or physician* or psycholog* or psychiatr* or pharmac* or nutritionist* or speech-language pathologist* or patient care team* or case manage* or liaison nurse* or collaborative practice* or collaborative care or nurse-led follow-up or interdisciplinary care or interdisciplinary team* or interdisciplinary care team* or multidimensional care or multidimensional team* or multidimensional care team* or multi dimensional care or multi dimensional team* or multi dimensional care team* or team care or multi agency working or inter agency working or multi professional working or interprofessional working or interprofessional team* or inter professional team* or multidisciplinary team* or multidisciplinary care team* or interdisciplinary treatment approach* or integrated care).tw. |
| 8 | 6 and 7 |
| 9 | 4 or 5 or 8 |
| 10 | Quality of life/ or (Quality of life or QoL).tw. |
| 11 | Accidental falls/ or fall*.tw. |
| 12 | Depression/ or depress*.tw. |
| 13 | exp Dementia/ or dementia*.tw. |
| 14 | exp Emergency service, hospital/ |
| 15 | 10 or 11 or 12 or 13 or 14 |
| 16 | exp Randomized controlled trials as topic/ or random allocation/ or exp controlled clinical trials as topic/ or exp clinical trials as topic/ or exp cohort studies/ or exp case-control studies/ or cross-sectional studies/ or (randomi* controlled trial* or RCT or RCTs).tw. |
| 17 | (Systematic review or meta-analysis or review or letter or case reports or guideline).pt. or (systematic review or meta-analysis or review).ti. |
| 18 | 16 not 17 |
| 19 | 3 and 9 and 15 and 18 |
| 20 | limit 19 to (dt=20100101-20200731 and humans and English) |
